# Supplementary material for: Machine Learning Accelerates Crystallization for Structure Determination
Source: Angew Chem Int Ed Engl. 2026 May 4;65(25):e1218503. doi: 10.1002/anie.1218503 (PMC13266958; doi:10.1002/anie.1218503)

# checkCIF/PLATON report

Structure factors have been supplied for datablock(s) lcz108

THIS REPORT IS FOR GUIDANCE ONLY. IF USED AS PART OF A REVIEW PROCEDURE FOR PUBLICATION, IT SHOULD NOT REPLACE THE EXPERTISE OF AN EXPERIENCED CRYSTALLOGRAPHIC REFEREE.

No syntax errors found. CIF dictionary Interpreting this report

# Datablock: lcz108

---

| | | | |
|---|---|---|---|
| Bond precision: | C-C = 0.0339 A | | Wavelength=1.54184 |
| Cell: | a=32.3294(8) | b=13.4850(4) | c=21.4483(5) |
| | alpha=90 | beta=99.988(2) | gamma=90 |
| Temperature: | 100 K | | |

| | Calculated | Reported |
|---|---|---|
| Volume | 9208.9(4) | 9208.9(4) |
| Space group | C 2 | C 1 2 1 |
| Hall group | C 2y | C 2y |
| Moiety formula | C117 H97 Ag9 F54 N18 O20, C15 H3 Ag3 F18 N6 [+ solvent] | C15 H3 Ag3 F18 N6, C18 H22 O5, 0.25[], 0.25[] |
| Sum formula | C132 H100 Ag12 F72 N24 O20 [+ solvent] | C33 H25 Ag3 F18 N6 O5 |
| Mr | 5004.81 | 1251.20 |
| Dx,g cm-3 | 1.805 | 1.805 |
| Z | 2 | 8 |
| Mu (mm-1) | 11.216 | 11.216 |
| F000 | 4864.0 | 4864.0 |
| F000’ | 4886.57 | |
| h,k,lmax | 40,16,26 | 40,16,26 |
| Nref | 18957[ 9899] | 15114 |
| Tmin,Tmax | 0.232,0.208 | 0.360,1.000 |
| Tmin’ | 0.149 | |

```
Correction method= # Reported T Limits: Tmin=0.360 Tmax=1.000
AbsCorr = MULTI-SCAN

Data completeness= 1.53/0.80        Theta(max)= 74.991

R(reflections)= 0.0820( 13493)          wR2(reflections)= 0.2499( 15114)

S = 1.146                   Npar= 1134
```

---

The following ALERTS were generated. Each ALERT has the format
**test-name_ALERT_alert-type_alert-level**.
Click on the hyperlinks for more details of the test.

---

**Alert level A**

```
PLAT973_ALERT_2_A Check Calcd Positive Resid. Density on      Ag01       2.28 eA-3
```

**Author Response: This Alert is due to presence of residual density in the presence of heavy metal atom (Ag).**

---

**Alert level B**

```
SHFSU01_ALERT_2_B  The absolute value of parameter shift to su ratio > 0.10
            Absolute value of the parameter shift to su ratio given   0.150
            Additional refinement cycles may be required.
```

**Author Response: We made several attempts to obtain a higher quality data set for this compound. However, due to solvent disorder and poor crystal quality, we were unable to lower the absolute value of parameter shift. Nevertheless, there is no doubt regarding the framework of the structure.**

```
PLAT080_ALERT_2_B Maximum Shift/Error ...........................       0.15 Why ?
```

**Author Response: We have tried to increase the number of fine-tuning rounds to solve this problem, but this is the best result we have achieved so far.**

```
PLAT234_ALERT_4_B Large Hirshfeld Difference C01F     --C017     .       0.30 Ang.
```

**Author Response: atoms from a modeled disordered moiety; relevant atomic sites have been checked and they were found to be consistent with the chemistry performed.**

```
PLAT342_ALERT_3_B Low Bond Precision on  C-C Bonds ..............    0.03392 Ang.
```

**Author Response: Disordered structure.**

```
PLAT420_ALERT_2_B D-H Bond Without Acceptor  O013     --H013     .     Please Check
```

**Author Response: The solvent molecules are highly disordered, which supposed to form hydrogen bond with O have been removed by the SQUEEZE routine in the PLATON software package.**

```
PLAT420_ALERT_2_B D-H Bond Without Acceptor  O01M     --H01M     .     Please Check
```

**Author Response: The solvent molecules are highly disordered, which supposed to form hydrogen bond with O have been removed by the SQUEEZE routine in the PLATON software package.**

---

**Alert level C**

```
PLAT234_ALERT_4_C Large Hirshfeld Difference Ag04     --N008     .       0.18 Ang.
```

**Author Response: atoms from a modeled disordered moiety; relevant atomic sites have been checked and they were found to be consistent with the chemistry performed.**

```
PLAT234_ALERT_4_C Large Hirshfeld Difference Ag07     --N00C     .       0.16 Ang.
```

**Author Response: atoms from a modeled disordered moiety; relevant atomic sites have been checked and they were found to be consistent with the chemistry performed.**

```
PLAT234_ALERT_4_C Large Hirshfeld Difference F00H     --C031     .       0.20 Ang.
```

**Author Response: atoms from a modeled disordered moiety; relevant atomic sites have been checked and they were found to be consistent with the chemistry performed.**

```
PLAT234_ALERT_4_C Large Hirshfeld Difference F01U     --C01R     .       0.24 Ang.
```

**Author Response: atoms from a modeled disordered moiety; relevant atomic sites have been checked and they were found to be consistent with the chemistry performed.**

PLAT234_ALERT_4_C Large Hirshfeld Difference F029 --C02Y . 0.18 Ang.

**Author Response: atoms from a modeled disordered moiety; relevant atomic sites have been checked and they were found to be consistent with the chemistry performed.**

PLAT234_ALERT_4_C Large Hirshfeld Difference O00W --C03I . 0.19 Ang.

**Author Response: atoms from a modeled disordered moiety; relevant atomic sites have been checked and they were found to be consistent with the chemistry performed.**

PLAT234_ALERT_4_C Large Hirshfeld Difference O00Z --C02T . 0.19 Ang.

**Author Response: atoms from a modeled disordered moiety; relevant atomic sites have been checked and they were found to be consistent with the chemistry performed.**

PLAT234_ALERT_4_C Large Hirshfeld Difference O013 --C02M . 0.22 Ang.

**Author Response: atoms from a modeled disordered moiety; relevant atomic sites have been checked and they were found to be consistent with the chemistry performed.**

PLAT234_ALERT_4_C Large Hirshfeld Difference O01K --C03I . 0.19 Ang.

**Author Response: atoms from a modeled disordered moiety; relevant atomic sites have been checked and they were found to be consistent with the chemistry performed.**

PLAT234_ALERT_4_C Large Hirshfeld Difference O01M --C02X . 0.19 Ang.

**Author Response: atoms from a modeled disordered moiety; relevant atomic sites have been checked and they were found to be consistent with the chemistry performed.**

PLAT234_ALERT_4_C Large Hirshfeld Difference C00R --C011 . 0.21 Ang.

**Author Response: atoms from a modeled disordered moiety; relevant atomic sites have been checked and they were found to be consistent with the chemistry performed.**

PLAT234_ALERT_4_C Large Hirshfeld Difference C011 --C01O . 0.19 Ang.

**Author Response: atoms from a modeled disordered moiety; relevant atomic sites have been checked and they were found to be consistent with the chemistry performed.**

PLAT234_ALERT_4_C Large Hirshfeld Difference C012 --C028 . 0.20 Ang.

**Author Response: atoms from a modeled disordered moiety; relevant atomic sites have been checked and they were found to be consistent with the chemistry performed.**

PLAT234_ALERT_4_C Large Hirshfeld Difference C11 --C03K . 0.22 Ang.

**Author Response: atoms from a modeled disordered moiety; relevant atomic sites have been checked and they were found to be consistent with the chemistry performed.**

PLAT234_ALERT_4_C Large Hirshfeld Difference C017 --C01X . 0.20 Ang.

**Author Response: atoms from a modeled disordered moiety; relevant atomic sites have been checked and they were found to be consistent with the chemistry performed.**

PLAT234_ALERT_4_C Large Hirshfeld Difference C035 --C03O . 0.24 Ang.

**Author Response: atoms from a modeled disordered moiety; relevant atomic sites have been checked and they were found to be consistent with the chemistry performed.**

PLAT234_ALERT_4_C Large Hirshfeld Difference F02S --C036 . 0.22 Ang.

**Author Response: atoms from a modeled disordered moiety; relevant atomic sites have been checked and they were found to be consistent with the chemistry performed.**

```
PLAT241_ALERT_2_C High   'MainMol' Ueq as Compared to Neighbors of       C01F Check
PLAT241_ALERT_2_C High   'MainMol' Ueq as Compared to Neighbors of       C01T Check
PLAT241_ALERT_2_C High   'MainMol' Ueq as Compared to Neighbors of       C03C Check
PLAT241_ALERT_2_C High   'MainMol' Ueq as Compared to Neighbors of       C888 Check
PLAT242_ALERT_2_C Low    'MainMol' Ueq as Compared to Neighbors of       O01L Check
PLAT242_ALERT_2_C Low    'MainMol' Ueq as Compared to Neighbors of       C023 Check
PLAT242_ALERT_2_C Low    'MainMol' Ueq as Compared to Neighbors of       C026 Check
PLAT242_ALERT_2_C Low    'MainMol' Ueq as Compared to Neighbors of       C028 Check
PLAT242_ALERT_2_C Low    'MainMol' Ueq as Compared to Neighbors of       C03N Check
PLAT242_ALERT_2_C Low    'MainMol' Ueq as Compared to Neighbors of       C02T Check
```

```
PLAT242_ALERT_2_C Low    'MainMol' Ueq as Compared to Neighbors of       C02X Check
PLAT309_ALERT_2_C Single Bonded Oxygen (C-O > 1.3 Ang) ..........       O00X Check
PLAT360_ALERT_2_C Short  C(sp3)-C(sp3) Bond  C01E     - C03M     .       1.39 Ang.
PLAT362_ALERT_2_C Short  C(sp3)-C(sp2) Bond  C1       - C023     .       1.40 Ang.
PLAT362_ALERT_2_C Short  C(sp3)-C(sp2) Bond  C034     - C03N     .       1.41 Ang.
PLAT363_ALERT_2_C Long   C(sp3)-C(sp2) Bond  C01E     - C01H     .       1.63 Ang.
PLAT911_ALERT_3_C Missing FCF Refl Between Thmin & STh/L=    0.600         56 Report
                0  8  0,   3  1  0,   4  0  0,  32  4  0,  33  5  0, -34  4  1,
               -1  1  1,   3  1  1,   7  1  1,  20  0  1,  32  4  1, -34  4  2,
               -4 16  2,  -2  0  2,   0  0  2,   1  1  2,   2  0  2,   7  7  2,
               31  5  2, -36  4  3, -34  4  3,  30  4  3,   0  0  4,  28  4  4,
              -36  4  5, -17  3  5,  20  0  5,  26  4  5,   0  0  6,   2  0  6,
                    (   26 More Missing: see the .ckf listing file)
PLAT913_ALERT_3_C Missing # of Very Strong Reflections in FCF ....          5 Note
                7  1  1,   2  0  2,   0  0  4,   0  0  6,   2  0  6,
PLAT915_ALERT_3_C No Flack x Check Done: Low Friedel Pair Coverage         60 %
PLAT934_ALERT_3_C Number of (Iobs-Icalc)/Sigma(W) > 10 Outliers ..          1 Check
              -28 -4 18,
PLAT972_ALERT_2_C Check Calcd Resid. Dens.  0.67Ang From Ag01           -1.57 eA-3
PLAT975_ALERT_2_C Check Calcd Resid. Dens.  0.91Ang From O00Z    .       1.06 eA-3
PLAT977_ALERT_2_C Check Negative Difference Density on H00Z      .      -0.37 eA-3
```

---

### Alert level G

```
PLAT003_ALERT_2_G Number of Uiso or U(i,j)  Restrained non-H-Atoms         63 Report
PLAT007_ALERT_5_G Number of Unrefined Donor-H Atoms .............           4 Report
              H00P  H00Z  H013  H01M
PLAT033_ALERT_4_G Flack x Value Deviates > 3.0 * Sigma from Zero .      0.121 Note
PLAT041_ALERT_1_G Calc. and Reported SumFormula    Strings  Differ     Please Check
              Calc: C132 H100 Ag12 F72 N24 O20
              Rep.: C33 H25 Ag3 F18 N6 O5
PLAT042_ALERT_1_G Calc. and Reported MoietyFormula Strings  Differ     Please Check
              Calc: C117 H97 Ag9 F54 N18 O20, C15 H3 Ag3 F18 N6
              Rep.: C15 H3 Ag3 F18 N6, C18 H22 O5, 0.25[], 0.25[]
PLAT045_ALERT_1_G Calculated and Reported Z Differ by a Factor ...      0.250 Check
PLAT072_ALERT_2_G SHELXL First  Parameter in WGHT  Unusually Large       0.15 Report
PLAT083_ALERT_2_G SHELXL Second Parameter in WGHT  Unusually Large      55.07 Why ?
PLAT171_ALERT_4_G The CIF-Embedded .res File Contains EADP Records          3 Report
PLAT177_ALERT_4_G The CIF-Embedded .res File Contains DELU Records         15 Report
PLAT178_ALERT_4_G The CIF-Embedded .res File Contains SIMU Records          1 Report
PLAT186_ALERT_4_G The CIF-Embedded .res File Contains ISOR Records         13 Report
PLAT188_ALERT_3_G A Non-default SIMU Restraint Value has been used     0.0200 Report
PLAT192_ALERT_3_G A Non-default DELU Restraint Value for SecondPar     0.0200 Report
PLAT192_ALERT_3_G A Non-default DELU Restraint Value for SecondPar     0.0200 Report
PLAT192_ALERT_3_G A Non-default DELU Restraint Value for SecondPar     0.0200 Report
PLAT192_ALERT_3_G A Non-default DELU Restraint Value for SecondPar     0.0200 Report
PLAT192_ALERT_3_G A Non-default DELU Restraint Value for SecondPar     0.0200 Report
PLAT192_ALERT_3_G A Non-default DELU Restraint Value for SecondPar     0.0200 Report
PLAT192_ALERT_3_G A Non-default DELU Restraint Value for SecondPar     0.0200 Report
PLAT192_ALERT_3_G A Non-default DELU Restraint Value for SecondPar     0.0200 Report
PLAT192_ALERT_3_G A Non-default DELU Restraint Value for SecondPar     0.0200 Report
PLAT192_ALERT_3_G A Non-default DELU Restraint Value for SecondPar     0.0200 Report
PLAT242_ALERT_2_G Low    'MainMol' Ueq as Compared to Neighbors of         C1 Check
PLAT242_ALERT_2_G Low    'MainMol' Ueq as Compared to Neighbors of       C01O Check
PLAT242_ALERT_2_G Low    'MainMol' Ueq as Compared to Neighbors of       C01R Check
PLAT242_ALERT_2_G Low    'MainMol' Ueq as Compared to Neighbors of       C020 Check
PLAT242_ALERT_2_G Low    'MainMol' Ueq as Compared to Neighbors of       C02V Check
```

```
PLAT242_ALERT_2_G Low    'MainMol' Ueq as Compared to Neighbors of       C02Y Check
PLAT242_ALERT_2_G Low    'MainMol' Ueq as Compared to Neighbors of       C031 Check
PLAT242_ALERT_2_G Low    'MainMol' Ueq as Compared to Neighbors of       C034 Check
PLAT242_ALERT_2_G Low    'MainMol' Ueq as Compared to Neighbors of       C038 Check
PLAT242_ALERT_2_G Low    'MainMol' Ueq as Compared to Neighbors of       C02E Check
PLAT242_ALERT_2_G Low    'MainMol' Ueq as Compared to Neighbors of       C030 Check
PLAT242_ALERT_2_G Low    'MainMol' Ueq as Compared to Neighbors of       C036 Check
PLAT303_ALERT_2_G Full Occupancy Atom H00P      with # Connections       2.00 Check
PLAT343_ALERT_2_G Unusual sp3      Angle Range in Main Residue for         C1 Check
PLAT343_ALERT_2_G Unusual sp3      Angle Range in Main Residue for       C030 Check
PLAT434_ALERT_2_G Short Inter HL..HL Contact F024     ..F02P     .       2.84 Ang.
                                              x,-1+y,z  =      1_545 Check
PLAT606_ALERT_4_G Solvent Accessible VOID(S) in  Crystal Structure          ! Info
PLAT720_ALERT_4_G Number of Unusual/Non-Standard Labels ..........        174 Note
              Ag01    Ag02    Ag03    Ag04    Ag05    Ag06    Ag07    N008
              N009    N00A    F00B    N00C    N00E    N00F    O00G    F00H
              F00I    C00J    H00J    F00K    N00L    F00M    F00N    F00O
              O00P    H00P    N00Q    C00R    H00R    N00S    C00T    F00U
              N00V    O00W    O00X    O00Y    O00Z    H00Z    C010    C011
              C012    H012    O013    H013    F014    C015    C016    F018
              C019    F01A    C01C    H01G    F01D    C01E    H01A    H01B
              C01F    C017    C02M    C01S    H01S    C03K    H03K    N01G
              C01H    F01I    F01J    O01K    O01L    O01M    H01M    F01N
              C01O    N01P    C01R    C01T    H01C    H01D    F01U    C01V
              C01W    H01W    C01X    C01Y    H01E    H01F    C01Z    H01Z
              C020    F021    C022    H022    C023    F024    F025    C026
              C027    H027    C028    H028    F029    F02A    C02B    H02F
              F02C    F02D    C02E    C02F    H02G    H02H    C02G    H02A
              H02B    F02H    F02I    C02J    C02K    C02L    H02L    F02N
              F02P    F02Q    F02S    C02U    H02I    H02J    C02V    C02W
              H02K    H02M    H02N    C02Y    C02Z    H02C    H02D    H02E
              C030    C031    C034    C035    C036    C037    H03G    H03H
              C038    F039    F03B    C03C    H03A    H03B    C03D    H03I
              H03J    C03F    H03C    H03D    C03H    H03L    H03M    C03I
              C03M    H03E    H03F    C03N    C03O    H03O    C03P    H03P
              C02T    C033    C01Q    C02R    H02R    C02X
PLAT791_ALERT_4_G Model has Chirality at C02L       (Sohncke SpGr)          S Verify
PLAT791_ALERT_4_G Model has Chirality at C03P       (Sohncke SpGr)          S Verify
PLAT860_ALERT_3_G Number of Least-Squares Restraints .............        304 Note
PLAT868_ALERT_4_G ALERTS Due to the Use of _smtbx_masks Suppressed          ! Info
PLAT883_ALERT_1_G Absent Datum for _atom_sites_solution_primary ..     Please Do !
PLAT910_ALERT_3_G Missing FCF Reflection(s) Below Theta(Min)[Deg]=       3.76 Note
                1  1  0,   2  0  0,  -2  0  1,   0  0  1,
PLAT912_ALERT_4_G Missing # of FCF Reflections Above STh/L=  0.600        179 Note
PLAT916_ALERT_2_G Hooft y and Flack x Parameter Values Differ by .       0.12 Check
PLAT933_ALERT_2_G Number of HKL-OMIT Records in Embedded .res File         53 Note
              -30 -4 12, -30 -4 13, -30 -4 14, -30 -4 15, -30 -4 16, -30 -4 17,
              -30 -2 13, -30 -2 15, -30 -2 16, -29 -5 12, -29 -5 13, -29 -5 16,
              -29 -5 17, -29 -5 18, -29 -3 19, -29 -3 20, -28 -6 16, -28 -6 18,
              -28 -4 10, -28 -4 11, -28 -4 12, -28 -4 17, -27 -7 13, -27 -5  9,
              -27 -5 11, -27 -5 12, -27 -5 13, -27 -5 15, -27 -5 16, -27 -5 18,
              -27 -5 19, -27 -5 20, -27 -5 21, -27 -3 22, -26 -6 13, -26 -6 14,
              -26 -6 18, -26 -6 20, -25 -7 14, -25 -7 15, -25 -7 19, -25 -5 20,
              -25 -5 21, -25 -5 22, -24 -8 16, -24 -6 21, -23 -7 18, -23 -7 19,
              -22 -8 18, -22 -8 19,
PLAT941_ALERT_3_G Average HKL Measurement Multiplicity ..........        2.7 Low
PLAT967_ALERT_5_G Note: Two-Theta Cutoff Value in Embedded .res ..      150.0 Degree
```

```
PLAT969_ALERT_5_G The 'Henn et al.' R-Factor-gap value ..........      4.876 Note
              Predicted wR2: Based on SigI**2  5.12 or SHELX Weight 21.81
PLAT978_ALERT_2_G Number C-C Bonds with Positive Residual Density.         0 Info
PLAT992_ALERT_5_G Repd & Actual _reflns_number_gt Values Differ by         2 Check
```

```
   1 ALERT level A = Most likely a serious problem - resolve or explain
   6 ALERT level B = A potentially serious problem, consider carefully
  40 ALERT level C = Check. Ensure it is not caused by an omission or oversight
  55 ALERT level G = General information/check it is not something unexpected

   4 ALERT type 1 CIF construction/syntax error, inconsistent or missing data
  46 ALERT type 2 Indicator that the structure model may be wrong or deficient
  19 ALERT type 3 Indicator that the structure quality may be low
  29 ALERT type 4 Improvement, methodology, query or suggestion
   4 ALERT type 5 Informative message, check
```

It is advisable to attempt to resolve as many as possible of the alerts in all categories. Often the minor alerts point to easily fixed oversights, errors and omissions in your CIF or refinement strategy, so attention to these fine details can be worthwhile. It is up to the individual to critically assess their own results and, if necessary, seek expert advice.

## Validation response form

Please find below a validation response form (VRF) that can be filled in and pasted into your CIF.

```
# start Validation Reply Form
_vrf_PLAT241_lcz108
;
PROBLEM: High   'MainMol' Ueq as Compared to Neighbors of       C01F Check
RESPONSE: ...
;
_vrf_PLAT242_lcz108
;
PROBLEM: Low    'MainMol' Ueq as Compared to Neighbors of       O01L Check
RESPONSE: ...
;
_vrf_PLAT309_lcz108
;
PROBLEM: Single Bonded Oxygen (C-O > 1.3 Ang) ..........       O00X Check
RESPONSE: ...
;
_vrf_PLAT360_lcz108
;
PROBLEM: Short  C(sp3)-C(sp3) Bond  C01E     - C03M     .       1.39 Ang.
RESPONSE: ...
;
_vrf_PLAT362_lcz108
;
PROBLEM: Short  C(sp3)-C(sp2) Bond  C1       - C023     .       1.40 Ang.
RESPONSE: ...
;
_vrf_PLAT363_lcz108
;
```

```
PROBLEM: Long   C(sp3)-C(sp2) Bond  C01E     - C01H     .       1.63 Ang.
RESPONSE: ...
;
_vrf_PLAT911_lcz108
;
PROBLEM: Missing FCF Refl Between Thmin & STh/L=    0.600         56 Report
RESPONSE: ...
;
_vrf_PLAT913_lcz108
;
PROBLEM: Missing # of Very Strong Reflections in FCF ....          5 Note
RESPONSE: ...
;
_vrf_PLAT915_lcz108
;
PROBLEM: No Flack x Check Done: Low Friedel Pair Coverage         60 %
RESPONSE: ...
;
_vrf_PLAT934_lcz108
;
PROBLEM: Number of (Iobs-Icalc)/Sigma(W) > 10 Outliers ..          1 Check
RESPONSE: ...
;
_vrf_PLAT972_lcz108
;
PROBLEM: Check Calcd Resid. Dens.  0.67Ang From Ag01          -1.57 eA-3
RESPONSE: ...
;
_vrf_PLAT975_lcz108
;
PROBLEM: Check Calcd Resid. Dens.  0.91Ang From O00Z    .       1.06 eA-3
RESPONSE: ...
;
_vrf_PLAT977_lcz108
;
PROBLEM: Check Negative Difference Density on H00Z      .      -0.37 eA-3
RESPONSE: ...
;
# end Validation Reply Form
```

---

**PLATON version of 17/09/2025; check.def file version of 17/09/2025**

---

# duplicate check

**No duplication found**

---

**Datablock lcz108** - ellipsoid plot

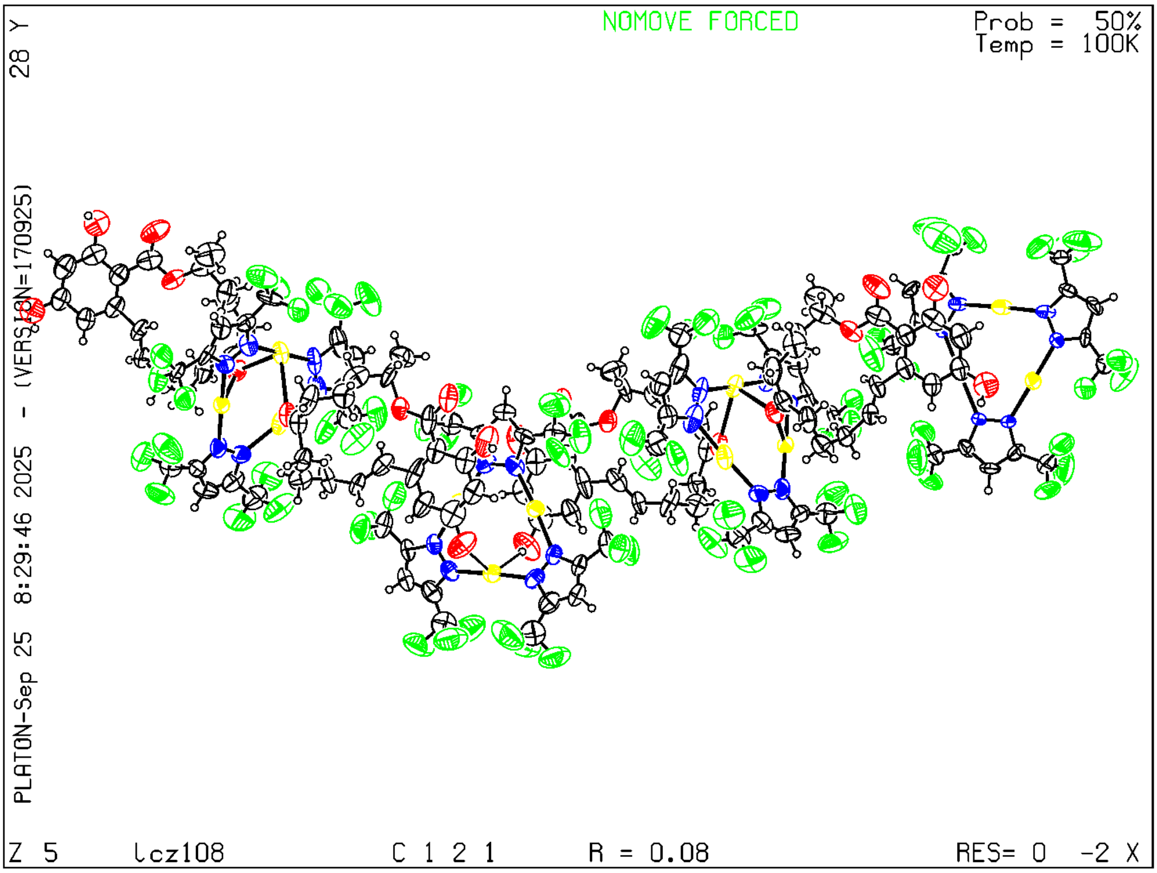

Supplement: Supplementary file 2 — Supporting File 2: anie72462‐sup‐0002‐DataFile.zip. [file ANIE-65-e1218503-s002.zip › supplementary_materials_crystallographic_data submit.zip!/cif_and_checkcif(1-114)/Ag3Pz3_108/Ag3Pz3_108_checkcif.pdf]
